# In vivo multiphoton imaging of immune cell dynamics

**DOI:** 10.1007/s00424-016-1882-x

**Published:** 2016-09-22

**Authors:** Takaharu Okada, Sonoko Takahashi, Azusa Ishida, Harumichi Ishigame

**Affiliations:** 1 Laboratory for Tissue Dynamics, RIKEN Center for Integrative Medical Sciences, Yokohama, Kanagawa 230-0045 Japan; 2 Graduate School of Medical Life Science, Yokohama City University, Yokohama, Kanagawa 230-0045 Japan; 3 PRESTO, Japan Science and Technology Agency, Kawaguchi, Saitama 332-0012 Japan

**Keywords:** Multiphoton microscopy, Immune response, Intravital imaging, Photoconversion

## Abstract

Multiphoton imaging has been utilized to analyze in vivo immune cell dynamics over the last 15 years. Particularly, it has deepened the understanding of how immune responses are organized by immune cell migration and interactions. In this review, we first describe the following technical advances in recent imaging studies that contributed to the new findings on the regulation of immune responses and inflammation. Improved multicolor imaging of immune cell behavior has revealed that their interactions are spatiotemporally coordinated to achieve efficient and long-term immunity. The use of photoactivatable and photoconvertible fluorescent proteins has increased duration and volume of cell tracking, even enabling the analysis of inter-organ migration of immune cells. In addition, visualization of immune cell activation using biosensors for intracellular calcium concentration and signaling molecule activities has started to give further mechanistic insights. Then, we also introduce recent imaging analyses of interactions between immune cells and non-immune cells including endothelial, fibroblastic, epithelial, and nerve cells. It is argued that future imaging studies that apply updated technical advances to analyze interactions between immune cells and non-immune cells will be important for thorough physiological understanding of the immune system.

**Electronic supplementary material:**

The online version of this article (doi:10.1007/s00424-016-1882-x) contains supplementary material, which is available to authorized users.

## Introduction

The immune system is comprised of diverse immune cell types. Different types of immune responses against various pathogens and environmental antigens need to employ different combinations of immune cells that interactively work together. Therefore, it is important to understand which types of immune cells are interacted with each other in specific locations in tissues at specific timings during each type of immune responses. Live imaging analysis with the 3D resolution at the cellular level using multiphoton laser excitation fluorescent microscopy (hereafter called multiphoton microscopy) provides vital information to understand in vivo cellular interactions. Earlier than in the immunology field, multiphoton microscopy was applied for live tissue imaging in the field of neuroscience in 1990s [10]. Real-time imaging of immune cell behavior in excised, re-aggregated, or in vivo tissues using multiphoton or single-photon laser microscopy was first reported in 2002 to 2003 [7, 45, 46, 67]. Since then, many techniques have been developed and applied to visualize various immunological events by multiphoton microscopy [16, 31].

Recently, for deeper understanding of complex but organized communications between immune cells, it is increasingly necessary to simultaneously track behavior of multiple immune cell types with high multicolor imaging performance. Another issue of multiphoton imaging is time and space limitations for tracking of highly motile immune cells, and photoactivatable and photoconvertible fluorescent proteins have been successfully utilized to extend these limits. Moreover, in order to obtain functional insights from imaging analysis, various reporters of cellular and molecular activities have been also employed for immune cell imaging. These reporters include not only genetically encoded calcium and apoptosis indicators, which have been used in neuroscience and developmental biology, but also a reporter of the MAP kinase activity, which was developed by cancer cell biologists. In this review, we introduce recent studies on immune cell dynamics and interactions utilizing the improved imaging techniques. Because there are recent excellent reviews on in vivo multiphoton imaging and imaging of immune cells, we mainly focus on the new studies and technical aspects that were not extensively described by the previous reviews [31, 60]. Then, we highlight recent studies that visualized interactions of immune cells with non-immune cells, and discuss future directions to study the regulation of immune responses and inflammation by the various systems of the body.

## Multicolor imaging of various immune cell types

In order to visually understand how cell migration and interactions underlie immune responses in vivo, it is helpful and often necessary to simultaneously get the picture of the tissue microenvironment where observation target events take place. For example, high affinity antibodies, which are an important arm of the immune system, are produced in a specialized structure, the germinal center formed in the lymphoid tissues during the immune response. Within the germinal center, antigen-specific B cells proliferate and mutate their antibody genes. B cells that have improved the affinity of their antibodies to antigens presented in the germinal center are positively selected by helper T cells to further proliferate and differentiate to antibody-secreting cells [1, 76]. For identifying germinal centers by in vivo imaging, it is useful to visualize the segregation of proliferating B cells and quiescent naïve B cells, which are not directly involved in ongoing immune responses [20, 35, 50]. Visualization of follicular dendritic cells, which present antigens in the form of immune complexes in germinal centers, can be also used to visually locate germinal centers [2, 77]. Thus, imaging with three colors or more is usually conducted to analyze migration and interactions of B cells and helper T cells in germinal centers.

Another example of the contribution of multicolor imaging can be found in recent research on the mechanism of long-term immunity by cytotoxic T cells, which are particularly important for viral and tumor immune responses. In this type of immune responses, again, helper T cells play an important role. As described above, some helper T cells migrate into germinal centers to control B cell responses, but others remain in the T cell zone of lymph nodes to help cytotoxic T cell activation and differentiation to long-lived memory T cells [6]. In most immune responses, T cell activation, expansion, and differentiation require interactions with antigen-presenting cells called dendritic cells. Within lymph nodes, there are various subsets of dendritic cells in different microenvironments [17, 25]. Generally, helper T cells are developed from naïve CD4^+^ T cells, and cytotoxic T cells from naïve CD8^+^ T cells after these naïve T cells are activated by dendritic cells. Several recent imaging studies showed that in the early phase of immune responses, naïve CD4^+^ T cells and naïve CD8^+^ T cells are activated by interactions with different subsets of dendritic cells in different locations [12, 18, 29, 36]. Interestingly, by comparing the results of these studies, it is suggested that subsets of dendritic cells involved in the early activation of CD4^+^ T cells and CD8^+^ T cells vary depending on the form of antigens and types of pathogens. In contrast, two of these studies using different viral infection models showed that after helper T cells and cytotoxic T cells are developed, both of them start interacting with a single subset of dendritic cells, which can be identified by the hallmark expression of a chemokine receptor XCR1 and presents antigens for both cytotoxic T cells and helper T cells [12, 29]. Through the “two-on-one” antigen-specific interactions, helper T cells potentiate function of the dendritic cell subset by providing cytokine and other signals, and cytotoxic T cells are maximally activated and become long-lived through the interaction with the potentiated XCR1^+^ dendritic cells [6].

**Fig. 1:**
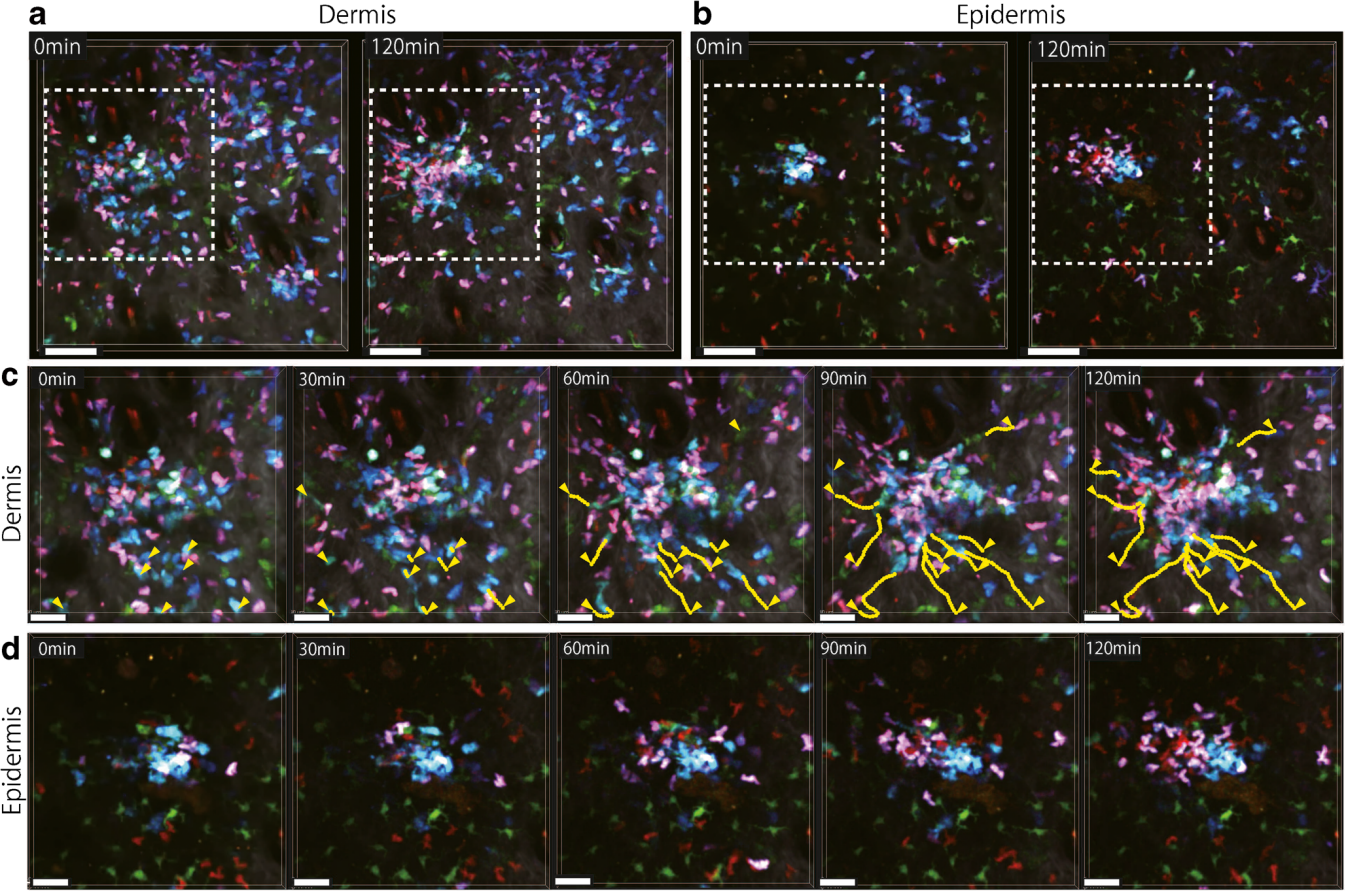
Multicolor imaging of dendritic cells and T cells in the mouse model of contact dermatitis by intravital multiphoton microscopy. Chicken ovalbumin-reactive T cell receptor transgenic CD4^+^ T cells expressing tdTomato plus EGFP (*pink to purple*) and CD8^+^ T cells expressing tdTomato (*red*) were transferred to an *Xcr1* ^*gfp/+*^ CD11c-YFP mouse for visualization of their interactions with XCR1^+^ dendritic cells (light blue) and other dendritic cells (green) [9]. The mouse was subcutaneously immunized in the flank with ovalbumin plus poly (I:C). Four days after immunization, the mice were then intradermally injected in the dorsum of foot with ovalbumin alone. Seven days later, the mouse was anesthetized, and the skin of the dorsum of foot was imaged on an inverted multiphoton microscope with four external detectors. Excitation wavelength was 910 nm. **a** Projection images of ten *z*-slices of the dermis (33–60-μm depth from the skin surface) at the beginning and end of the 2-h recording. *Scale bar*: 80 μm. Collagen fibers (*white*) were also visualized by second harmonic generation. **b** Projection images of four *z*-slices of the epidermis (12–21-μm depth from the skin surface) at the same *x-y* position as **a**. The scattered epidermal dendritic cells in green are mostly Langerhans cells. **c**, **d** Time-lapse images of the region indicated by *dotted lines* in **a** and **b**. *Scale bar*: 40 μm. *Yellow lines* in **c** are paths of dendritic cell migration tracked every minute. *Yellow arrowheads* indicate starting positions of the tracks

Differentiated helper T cells and cytotoxic T cells leave the lymph node to enter other tissues in which antigens and pathogens infiltrated. Then, they promote immune responses and inflammation in the tissues by interacting with local dendritic cells. An imaging study described that in the antigen-induced contact dermatitis model, dermal macrophages draw in dermal dendritic cells to form distinct clusters. Skin-infiltrated antigen-specific T cells swarm around and interact with the clustered dendritic cells, forming intense inflammation sites, which are suggested to be a cause of the eczematous vesicle formation [52]. Improved multicolor imaging shows that the dendritic cell clusters contain both XCR1^+^ and XCR1^−^ dendritic cells, and that both helper T cells and cytotoxic T cells swarm around the clusters (Fig. 1a, c; and Movie S1). Interestingly, the apex of some of the dendritic cell clusters seems to stick up into the epidermis, and helper T cells and cytotoxic T cells swarming in the dermis are observed to enter the epidermis at the apex of the dendritic cell clusters (Fig. 1b, d; and Movie S2). Some of these T cells, especially cytotoxic T cells, may stay in the epidermis to become tissue-resident memory T cells in preparation for future invasions [26].

In addition to the diversity of immune cells involved in immune responses in terms of their lineages and differentiation states, extreme diversity exists in the clonality of antigen receptor gene rearrangement in B and T cells. To visually estimate the clonality of B cells involved in each of germinal centers, a recent study utilized a multicolor imaging method based on “Brainbow,” which was originally developed for analysis of neural circuits and was also applied for fate-mapping analysis of epithelial stem cells and cells in the immune system such as Langerhans cells and follicular dendritic cells [19, 32, 65, 70]. By combining the imaging method with sequencing of the immunoglobulin genes of individual B cells from each germinal center, the study showed that B cell competition to achieve affinity maturation progressed in various manners in individual germinal centers in the same lymph node [70].

## Longitudinal tracking of immune cells

Immune responses usually take days or longer from the onset to come to the peak, and weeks or longer to wane. In order to interpret the results of immune cell migration and interactions and understand their roles in immune responses, it is often important to identify and analyze imaged cells a day or more after their behavior of interest is observed, either by continuously tracking them or by labeling them during imaging. Although continuous intravital imaging over a day is feasible to see changes occurring in the particular part of tissues [52], it is usually difficult to continuously track individual motile cells within limited imaging volumes for more than an hour. Therefore, labeling cells of interest during imaging for later analysis is an attractive approach. Photoactivatable fluorescent proteins such as PA-GFP [54] or photoconvertible ones like Kaede [3] and KikGR [74] enable light-induced labeling of target cells during imaging. Usually, photoactivation and photoconversion of these photochromic fluorescent proteins are performed by irradiation with intense violet light. However, this single-photon irradiation method lacks spatial resolution in the direction of travel of irradiation light (usually the tissue-depth direction). In contrast, multiphoton irradiation at ∼720–840 nm allows photoactivation or photoconversion of PA-GFP, Kaede, or KikGR in a microscopically defined 3D volume to specifically label cells of interest [8, 61, 77]. By optimizing the multiphoton irradiation method, the destination of B cells and helper T cells, which had been observed in specific anatomical locations in the lymph node at the time of irradiation, was analyzed several hours to a day later [62, 68, 77].

In most of the previous studies, mice expressing PA-GFP, Kaede, or KikGR ubiquitously in the whole body were used for flow cytometric analysis after irradiation or as donors of transplantable immune cell types [62, 68, 72, 73, 77]. However, mice that express the photochromic proteins in specific subsets of immune cells have been also generated [36]. The abovementioned XCR1^+^ dendritic cells in the lymph node are actually a mixture of a population that develops in the lymph node and a population that develops in other organs including the skin and then migrates to the lymph node. By irradiating the skin of a knock-in mouse strain harboring the KikGR gene inserted in the *Xcr1* locus, these two populations of XCR1^+^ dendritic cells in the lymph node can be distinguished by in vivo imaging to analyze their interactions with T cells [36].

**Fig. 2:**
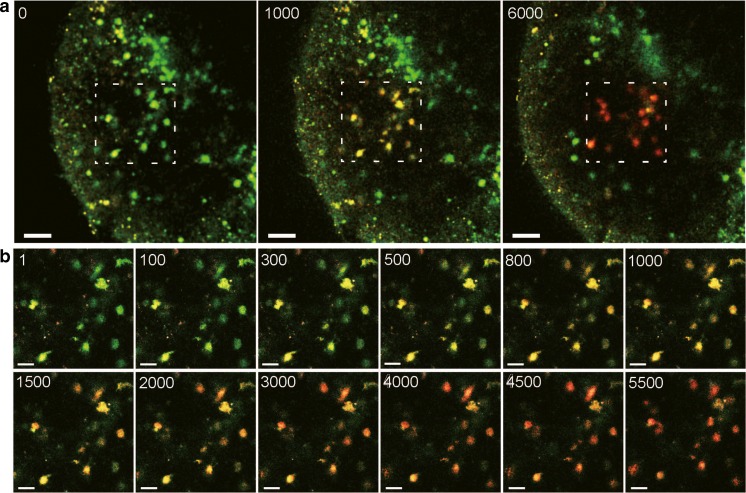
Multiphoton photoconversion of KikGR-expressing cells in the ex vivo lymph node. An inguinal lymph node was excised from an *Xcr1* ^*KikGR/+*^ mouse [36], and imaged and irradiated by multiphoton microscopy. **a** Single *xy*-slice images of the lymph node. Excitation wave length was 800 nm. The *numbers* indicate the number of times of 840-nm irradiation in the dotted line region before each image was recorded. *Scale bar*: 50 μm. **b** Single *xy*-slice images of the dotted line region in **a**. The images were recorded during 840-nm irradiation. The image size was 512 × 512 pixels, and laser dwell time during each *xy* scan was about 1.6 μs/pixel. *Scale bar*: 20 μm

When photoactivation or photoconversion is induced in cells within microscopically defined volumes by multiphoton irradiation, it is important to carefully determine the optimal laser intensity and exposure time for each experiment. A continuous exposure with high-power laser would damage cells and tissues, whereas a brief exposure with moderate-power laser would only photoactivate or photoconvert a small number of the fluorescent proteins. In the studies that used Kaede mice, two-photon irradiation with a moderate-power laser was repeated several thousand times to complete photoconversion of cells in the lymph node [8, 68]. KikGR-expressing dendritic cells in the lymph node can be photoconverted by using a similar irradiation protocol (Fig. 2). Specific photoconversion of motile target cells may require cell tracking during repeated irradiation or transient reduction of motility by lowering tissue temperature during repeated irradiation.

## Fluorescent indicators to visualize immune cell activities

For understanding the roles for immune cell migration and interactions, simultaneous visualization of cellular states and function by using molecular probes provides important information. The probes are either small molecule fluorescent indicators or fluorescent protein-based biosensors. Small molecule fluorescent indicators have been used to detect events such as changes in intracellular calcium concentrations [57, 79] and the generation of reactive oxygen species [11] in immune cells. Selective visualization of molecular events associated with endogenous target cells with small molecule fluorophores often requires ingenious in vivo labeling methods. For example, a recent study developed a pH-activatable small molecule probe that is fixed on the bone surface after the in vivo administration and reported intravital imaging of the bone resorption activity of osteoclasts [42].

Fluorescent protein-based biosensors have the advantages that they can be expressed selectively in target cells and are not diluted in proliferating cells. Intracellular calcium increases [39, 40, 51, 63, 71, 83], nuclear translocation of the transcription factor, nuclear factor of activated T cells (NFAT) [41, 43, 56], and caspase 3 activation during apoptosis [15] were visualized in T cells or B cells in vivo by using biosensors. There are two types of biosensors available to monitor intracellular calcium increases. One is to detect Förster resonance energy transfer (FRET) as changes in either the acceptor-to-donor fluorescence intensity ratio or the donor fluorescence lifetime [39, 40, 51, 64, 71, 80, 82, 83]. The other is to simply detect changes in the fluorescence intensity of biosensors [14, 63, 80]. Both types of calcium biosensors have been recently used to visualize calcium increases in helper T cells interacting with B cells in germinal centers. These studies have suggested that productive interactions with B cells induce calcium increases in helper T cells to enhance signaling for positive selection of the B cells [40, 63].

FRET biosensors have been also developed to monitor the activities of small GTPases and various kinases that are important for intracellular signaling in diverse types of cells [4, 37, 82]. Among them, transgenic mouse lines ubiquitously expressing biosensors for ERK and PKA were recently established and utilized for in vivo multiphoton imaging [27, 33]. As for immune cell imaging, the ERK activity was monitored in granulocytes during their extravasation or accumulation to damaged tissue sites [33, 48, 49].

## Imaging of interactions between immune cells and non-immune cells

Development, differentiation, and function of immune cells are dependent on and regulated by tissue microenvironment composed of non-immune cells. For instance, immigration of immune cells from the blood flow to the parenchyma of specific organs requires their interactions with diverse types of endothelial cells. For intravital imaging of non-immune cells in lymph nodes, transgenic mice ubiquitously expressing GFP were lethally irradiated and transplanted with bone marrow cells from non-fluorescent mice [5] or mice with the CFP transgene [47]. Using such bone marrow chimeric mice, lymphocyte migration through the endothelium of high endothelial venules to the lymph node parenchyma was imaged with multiphoton microscopy [5, 47]. The study also visualized the network of fibroblastic reticular cells as the tissue framework for lymphocyte migration in the lymph node parenchyma [5]. Lymphocyte emigration from the lymph node parenchyma into the lymphatic sinuses was also studied by multiphoton microscopy. In these studies, the lymphatic endothelium was visualized by administration of a fluorescent dye-labeled antibody against Lyve-1, the surface marker of lymphatic endothelial cells [22, 23], or a fluorescent dye-labeled lectin [78]. In addition, several lymphatic reporter mouse strains have been developed [28]. In the skin, the interaction between myeloid cells and pericytes around arterioles and capillaries in the inflammatory conditions was analyzed by intravital microscopy [66].

In the barrier tissues such as the intestine and skin, immune cell interactions with epithelial cells are pivotal for antigen transport and pathogen recognition. An imaging study showed that goblet cells, an epithelial cell type that secretes mucus into the gut lumen, take in soluble antigens from the lumen and pass them to a subset of dendritic cells in the lamina propria of the small intestinal villi [44]. Another study visualized that lamina propria dendritic cells vigorously stick out and retract their processes between epithelial cells to capture pathogens from the lumen [13]. More recently, dynamics of two suppressive T cell types in the intestine, which are believed to be important for the regulation of immune responses and inflammation, were visualized. One subset is regulatory T cells that are found mainly in the lamina propria, and the other is called intraepithelial lymphocytes (IELs). It was suggested from the imaging and other experiments that regulatory T cells that moved from the lamina propria to the epithelium were converted to IELs in a microbiota-dependent fashion. These T cells might occasionally migrate between epithelial cells to briefly poke out to the luminal side [69]. Although it is unclear how dendritic cell processes and T cells could make it through the tight junctions between epithelial cells, these events might be associated with dynamics of epithelial cells and tight junctions [24]. In the skin epidermis, when Langerhans cells, a subset of dendritic cells located in the epidermis, stick out their processes through the tight junctions between keratinocytes to sample external antigens, local remodeling of the tight junctions takes place to allow the penetration by processes of Langerhans cells [38]. The dynamics of this remodeling process, however, has not been analyzed by intravital microscopy.

Regulation of the immune system and inflammation by the peripheral nervous system has been studied for a long time, and is beginning to recapture a great amount of attention [53, 75]. In pharmacologically induced murine dermatitis that resembles human psoriasis, nociceptive sensory neurons were reported to promote the production of proinflammatory cytokine IL-23 by dermal dendritic cells, and this study visualized contacts of dendritic cells with sensory nerve fibers by intravital microscopy [58]. A different study also showed that in anti-fungal immune response skin nociceptors stimulate a dermal dendritic cell subset with a neuropeptide CGRP to promote the IL-23 production, although this study did not perform imaging analysis [34]. In the small intestine, the close association of muscularis macrophages with active myenteric plexus nerves was visualized by using a calcium biosensor. The study concluded that upon bacterial infection, enteric sympathetic nerves release norepinephrine in the myenteric plexus to induce tissue-protective functions of muscularis macrophages that express β2 adrenergic receptors [14]. On the other hand, immune cells can produce detrimental effects on neuronal cells in autoimmune diseases. Intravital imaging of the brainstem using a cell death indicator and a calcium biosensor showed the degeneration process of neurons contacted by proinflammatory helper T cells in the mouse models of multiple sclerosis [64]. The degeneration and regeneration processes were also analyzed by imaging of the explanted spinal cord from mice in which a multiple sclerosis-like disease was induced by the viral infection and neural precursor cells were transplanted [21].

## Future perspectives

As described above, in vivo immune cell dynamics has been analyzed by using the evolving techniques of multicolor visualization, light-induced cell labeling, and monitoring of cellular activities. In order to further improve the physiological understanding of the immune system, future imaging studies need to analyze interactions of the immune system with the other systems in the body by exploiting all of these cutting-edge techniques. For example, simultaneous multicolor visualization of three-way interactions between immune cells, epithelial cells, and nerves in the barrier tissue will likely provide a novel insight into the regulation of inflammation. Intravital imaging analysis of various other cell types such as glial cells [30], whose importance in control of immune cell function is newly discovered, will be also important. In addition, the diversity of epithelial cells, peripheral neurons, glial cells, endothelial cells, and fibroblastic cells need to be clarified in future imaging studies. As for the immune cell diversity, newly discovered important cell types such as various innate lymphoid cells need to be imaged together with other immune cells and non-immune cells [30, 55, 59]. Moreover, activities of immune cells and other cells such as neurons need to be simultaneously monitored to understand functional significance of their interactions. To analyze outcomes in immune cells of their interactions with neurons, optogenetic stimulation of specific neuronal subsets will be also useful [81]. Even if immune cells show outcomes to be analyzed only after more than a day post-interaction, light-induced labeling during the interaction may enable analysis of such outcomes. These approaches will be valuable for exploring the regulation of inflammation not only in the skin and intestine but also in many other tissues of the body.

## Electronic supplementary material

Below is the link to the electronic supplementary material.

Movie S1 Associated with Fig. 1A. (MOV 6976 kb)

Movie S2 Associated with Fig. 1B. (MOV 3668 kb)
